# Supplementary figures and images for: Multimodal scene recognition using semantic segmentation and deep learning integration
Source: PeerJ Comput Sci. 2025 May 14;11:e2858. doi: 10.7717/peerj-cs.2858 (PMC12192964; doi:10.7717/peerj-cs.2858)

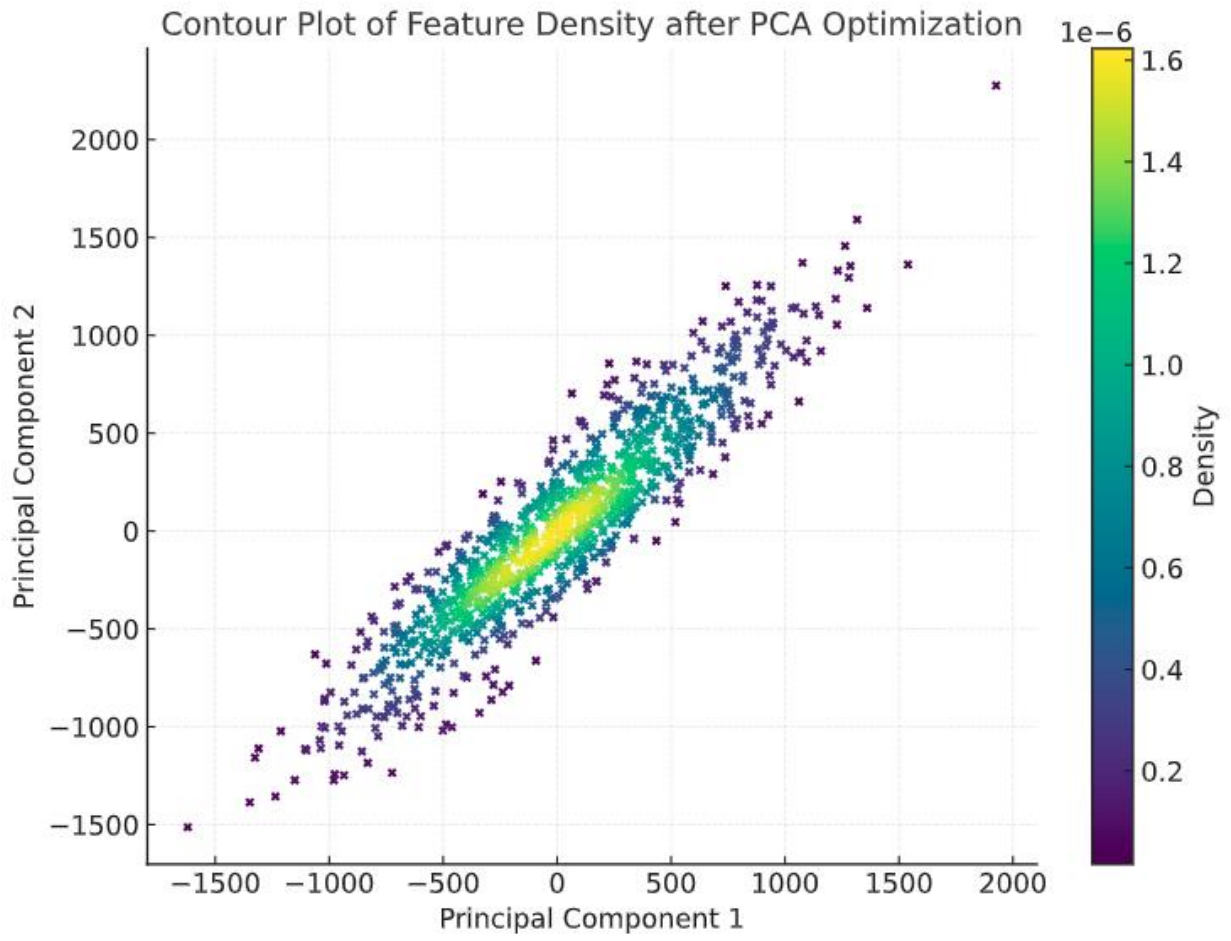

Supplement: Supplemental Information 1 [file peerj-cs-11-2858-s001.pdf]

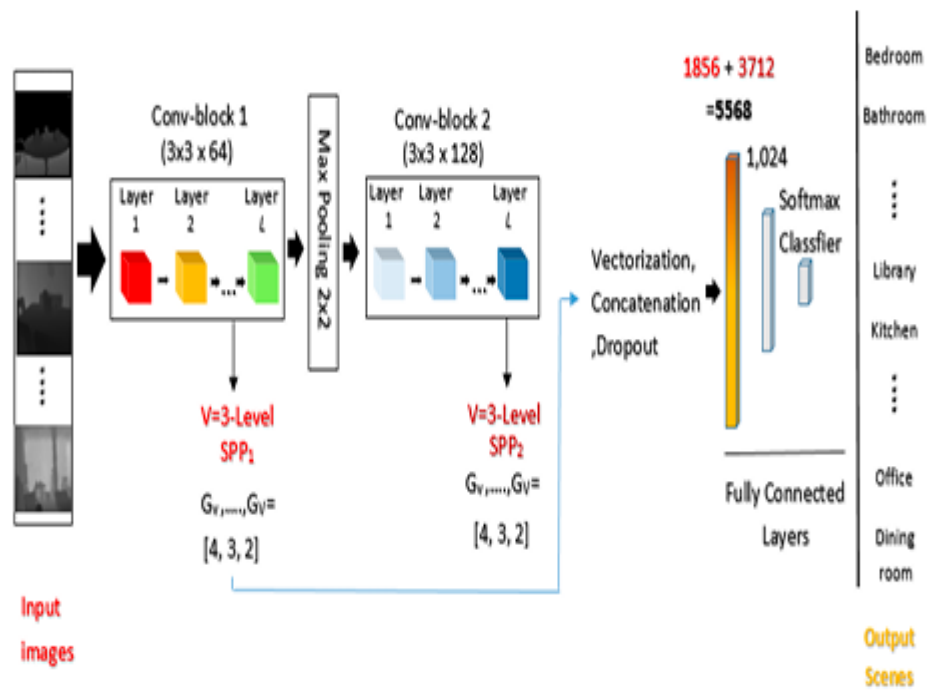

Multimodal Scene Classification Framework

Supplement: Supplemental Information 2 [file peerj-cs-11-2858-s002.pdf]

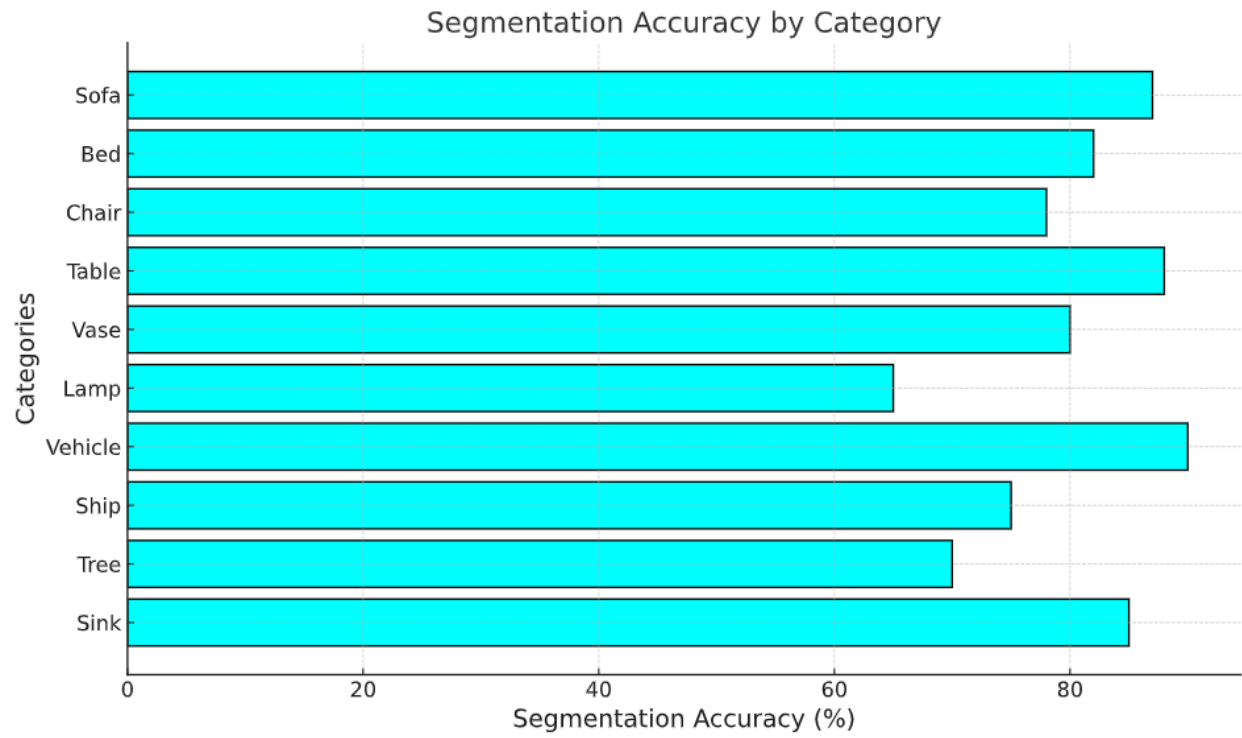

Supplement: Supplemental Information 3 [file peerj-cs-11-2858-s003.pdf]

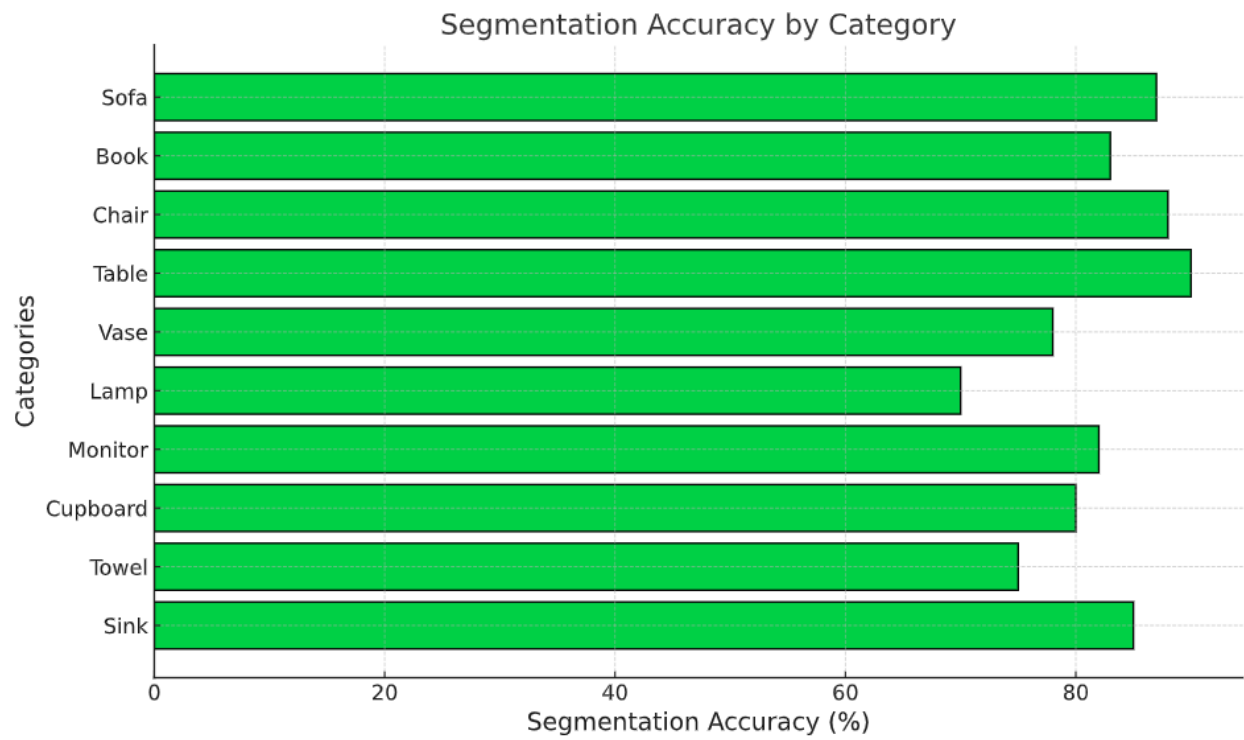

Supplement: Supplemental Information 4 [file peerj-cs-11-2858-s004.pdf]
