# Supplementary material for: Multimodal scene recognition using semantic segmentation and deep learning integration
Source: PeerJ Comput Sci. 2025 May 14;11:e2858. doi: 10.7717/peerj-cs.2858 (PMC12192964; doi:10.7717/peerj-cs.2858)
Supplement: Supplemental Information 5 [file peerj-cs-11-2858-s005.pdf]

```python
import numpy as np
import cv2
import pcl
import skimage
import torch
import torch.nn as nn
import torch.optim as optim
import torch.nn.functional as F
import torchvision.transforms as transforms
import torchvision.models as models
from sklearn.decomposition import PCA
from skimage.segmentation import felzenszwalb
from pydensecrf import densecrf as dcrf
from pydensecrf.utils import unary_from_labels
# Preprocessing Function
def preprocess_image(image):
    """Apply Gaussian Blur, normalization, and edge detection"""
    image_blurred = cv2.GaussianBlur(image, (5, 5), 0)
    image_normalized = cv2.normalize(image_blurred, None, 0, 255, cv2.NORM_MINMAX)
    edges = cv2.Canny(image_normalized.astype(np.uint8), 100, 200)
    return edges
# Segmentation using Felzenszwalb + CRF
def segment_image(image):
    """Apply Felzenszwalb segmentation and CRF for refinement"""
    segments_fz = felzenszwalb(image, scale=100, sigma=0.5, min_size=50)
    labels = segments_fz.astype(np.int32)
    d = dcrf.DenseCRF2D(image.shape[1], image.shape[0], np.max(labels) + 1)
    unary = unary_from_labels(labels, np.max(labels) + 1, gt_prob=0.7)
    d.setUnaryEnergy(unary)
```

```
    pairwise_bilateral = dcrf.create_pairwise_bilateral(sdims=(10, 10), schan=(20,), img=image, chdim=2)
    d.addPairwiseEnergy(pairwise_bilateral, compat=10)
    Q = d.inference(5)
    refined_labels = np.argmax(Q, axis=0).reshape(image.shape[:2])
    return refined_labels
```

# Feature Extraction: Voxel Grid Representation

```
def voxel_grid_from_depth(depth_image, voxel_size=0.05):
    """Convert depth image to a voxel grid representation"""
    cloud = pcl.PointCloud()
    points = np.array([np.random.rand(3) for _ in range(1000)], dtype=np.float32)
    cloud.from_array(points)
    sor = cloud.make_voxel_grid_filter()
    sor.set_leaf_size(voxel_size, voxel_size, voxel_size)
    voxel_cloud = sor.filter()
    return voxel_cloud.to_array()
```

# Feature Extraction: Surface Normals

```
def compute_surface_normals(depth_image):
    """Compute surface normals from a depth image"""
    sobelx = cv2.Sobel(depth_image, cv2.CV_64F, 1, 0, ksize=5)
    sobely = cv2.Sobel(depth_image, cv2.CV_64F, 0, 1, ksize=5)
    normals = np.sqrt(sobelx**2 + sobely**2)
    return normals
```

# Feature Optimization using PCA

```
def optimize_features(features):
    """Reduce dimensionality using PCA"""
    pca = PCA(n_components=50)
    return pca.fit_transform(features)
```

```python
# CNN Model with SPP Layer
class SPPNet(nn.Module):
    def __init__(self, num_classes=14):
        super(SPPNet, self).__init__()
        self.backbone = models.resnet18(pretrained=True)
        self.backbone.fc = nn.Identity()
        self.spp = nn.AdaptiveAvgPool2d((1, 1))
        self.fc = nn.Linear(512, num_classes)

    def forward(self, x):
        x = self.backbone(x)
        x = self.spp(x.unsqueeze(-1)).squeeze()
        x = self.fc(x)
        return F.log_softmax(x, dim=1)

# Training Function
def train_model(model, train_loader, criterion, optimizer, epochs=10):
    """Train CNN-SPP model"""
    model.train()
    for epoch in range(epochs):
        running_loss = 0.0
        for inputs, labels in train_loader:
            inputs, labels = inputs.to(device), labels.to(device)
            optimizer.zero_grad()
            outputs = model(inputs)
            loss = criterion(outputs, labels)
            loss.backward()
            optimizer.step()
            running_loss += loss.item()
```

```python
        print(f"Epoch {epoch+1}, Loss: {running_loss/len(train_loader)}")

# Main Execution
if __name__ == "__main__":
    # Load and preprocess image
    image = cv2.imread('sample_image.jpg', 0)
    processed_image = preprocess_image(image)
    segmented_image = segment_image(processed_image)

    # Extract features
    voxel_features = voxel_grid_from_depth(segmented_image)
    surface_normals = compute_surface_normals(segmented_image)

    # Optimize features
    combined_features = np.hstack((voxel_features, surface_normals))
    optimized_features = optimize_features(combined_features)

    # Initialize CNN model
    device = torch.device("cuda" if torch.cuda.is_available() else "cpu")
    model = SPPNet(num_classes=15).to(device)

    # Define loss and optimizer
    criterion = nn.CrossEntropyLoss()
    optimizer = optim.Adam(model.parameters(), lr=0.001)

    # Train model (assuming train_loader is defined)
    # train_model(model, train_loader, criterion, optimizer, epochs=100)
```
