# Supplementary material for: Multimodal scene recognition using semantic segmentation and deep learning integration
Source: PeerJ Comput Sci. 2025 May 14;11:e2858. doi: 10.7717/peerj-cs.2858 (PMC12192964; doi:10.7717/peerj-cs.2858)
Supplement: Supplemental Information 6 [file peerj-cs-11-2858-s006.pdf]

TECHNOLOGY INFRASTRUCTURE

The procedure was carried out using a Windows 10 computer that had an Intel Core i7 processor clocked at 3.60 GHz, an Nvidia Tesla K80: 2496 CUDA cores, 16 GB RAM. Python 3.6 and the Keras API were used in the model's development for both training and construction.
