# Supplementary material for: Multimodal scene recognition using semantic segmentation and deep learning integration
Source: PeerJ Comput Sci. 2025 May 14;11:e2858. doi: 10.7717/peerj-cs.2858 (PMC12192964; doi:10.7717/peerj-cs.2858)
Supplement: Supplemental Information 7 [file peerj-cs-11-2858-s007.pdf]

Multimodal Scene Recognition using Depth-Aware Segmentation and CNN-SPP

Overview

This work presents a new environment perception deep-learning network, following the multimodal approach, where depth and RGB images are used as input to increase the indoor scene segmentation and recognition. It uses depth-aware segmentation to overcome this problem as well as CNN with Spatial Pyramid Pooling (SPP) especially in occluded and low light conditions. They evaluate it on benchmark dataset including NYUD-v2 and RGB-D Scene where it reaches accurate results in segmenting and recognizing indoor scenes.

Table of Contents

1. Project Setup
2. Data Preparation
3. Code Explanation
4. Implementation Steps

Project Setup

Prerequisites

- **Python 3.6**
- **Libraries**: Install the required libraries by running:

```
pip install torch torchvision opencv-python numpy scipy matplotlib
```

System Requirements

This code has been tested on Windows 10 with the following configuration:

- **CPU**: Intel Core i7
- **RAM**: 16 GB

For optimal performance, consider using a machine with higher specifications.

Data Preparation

The NYUD-v2 and RGB-D Scene datasets need to be downloaded freely available online and put in a folder called `Depth`. These images are taken directly and hence paths should match the requirements in the code. If required, then modify the ***load_depth_data*** function accordingly.

Code Explanation

The model consists of several key components, each implemented within the provided code:

1. **Preprocessing**: The preprocessing function ***preprocess_depth_data*** applies Gaussian Blur to reduce noise while preserving structural details, normalizes the image to a range of [0, 255], detects edges using the Canny algorithm, computes the gradient magnitude to enhance edges, and performs morphological closing to ensure smooth, continuous contours for segmentation.
2. **Falzenwalb's with CRF Segmentation**
   - **segments_fz** splits the image into segments by treating pixels as nodes in a graph, connecting adjacent pixels based on color variance and spatial proximity, with the scale parameter controlling the sensitivity to color variance (larger values create larger segments)
   - The **DenseCRF model** refines the segmentation from Felzenszwalb by using it as initial labels, applying pairwise potentials to smooth boundaries and reduce noise, with bilateral filters accounting for spatial closeness and color similarity.
3. **Feature Extraction**
   - **Voxel Grid Representation**: The `create_voxel_grid` function takes a depth image and creates a voxel grid. Each voxel represents a 3D space segment and stores points from the depth image.
   - **Point Cloud Generation**: The `depth_to_point_cloud` function transforms 2D pixel coordinates into 3D coordinates, creating a point cloud by mapping each depth pixel to 3D space using camera intrinsics (focal lengths and optical center).
   - **Surface Normals Calculation**: The `compute_surface_normals` function estimates surface normals using nearest neighbors within a radius. These normals help to understand object orientation and geometry.
4. **Feature Optimization**

   The `PCA` class from `sklearn.decomposition` is used to reduce the data's dimensionality from 10 to 2, while retaining as much variance as possible. The principal components are derived as eigenvectors of the covariance matrix of the dataset.
5. **Scene Classification with CNN-SPP**:
   - *CRFSceneClassifier* integrates information from ELM, Vision Transformer features, and depth data to classify the overall scene context using a CRF.

Implementation Steps

Step 1: Preprocess the Images

Run the preprocessing function to prepare the RGB and depth images:

```
processed_depth_image = preprocess_depth_image('path_to_depth_image')
```

Step 2: Segment the Image

Use the Felzenszwalb's algorithm and Conditional Random Fields (CRF) for segmentation.

```
segments_fz, refined_segments = segment_with_felzenszwalb_and_crf('path_to_image')
```

Step 3: Feature Extraction and optimization

```
voxel_grid = create_voxel_grid(segmented_image)

point_cloud = depth_to_point_cloud(segmented_image, fx, fy, cx, cy)

normals = compute_surface_normals(point_cloud)

features = voxel_grid, Point_cloud, normals(segmented_image)

pca_features = apply_pca(features)
```

Step 4: Classification with CNN_SPP

- Use the extracted features to classify scene:

```
model = CNN_SPP_Model(num_classes=10)

predictions = model(pca_features)
```

Step 5: Save and Load Model Parameters

To save the trained model:

```
torch.save(model.state_dict(), 'cnn_spp_model.pth')
```

To load the model for further evaluation:

```
model = CNN_SPP_Model(num_classes=10)

model.load_state_dict(torch.load('cnn_spp_model.pth'))

 model.eval()
```
